# Comparative Transcriptomic Studies on a Cadmium Hyperaccumulator *Viola baoshanensis* and Its Non-Tolerant Counterpart *V. inconspicua*

**DOI:** 10.3390/ijms20081906

**Published:** 2019-04-17

**Authors:** Haoyue Shu, Jun Zhang, Fuye Liu, Chao Bian, Jieliang Liang, Jiaqi Liang, Weihe Liang, Zhiliang Lin, Wensheng Shu, Jintian Li, Qiong Shi, Bin Liao

**Affiliations:** 1 State Key Laboratory of Biocontrol, School of Life Sciences, Sun Yat-Sen University, Guangzhou 510275, China; shuhy@mail2.sysu.edu.cn (H.S.); liufy6@126.com (F.L.); linzhliang@126.com (Z.L.); 2 School of Biosciences and Biopharmaceutics, Guangdong Province Key Laboratory for Biotechnology Drug Candidates, Guangdong Pharmaceutical University, Guangzhou 510006, China; zhangj80@aliyun.com (J.Z.); liangjq_phil@163.com (J.L.); TTlweihe@163.com (W.L.); 3 Shenzhen Key Lab of Marine Genomics, Guangdong Provincial Key Lab of Molecular Breeding in Marine Economic Animals, BGI Academy of Marine Sciences, BGI Marine, BGI, Shenzhen 518083, China; bianchao@genomics.cn; 4 School of Life Sciences, South China Normal University, Guangzhou 510631, China; liangjl@m.scnu.edu.cn (J.L.); shuws@mail.sysu.edu.cn (W.S.); lijtian@mail.sysu.edu.cn (J.L.)

**Keywords:** Cadmium, hyperaccumulator, *Viola baoshanensis*, transcriptome, detoxification

## Abstract

Many *Viola* plants growing in mining areas exhibit high levels of cadmium (Cd) tolerance and accumulation, and thus are ideal organisms for comparative studies on molecular mechanisms of Cd hyperaccumulation. However, transcriptomic studies of hyperaccumulative plants in Violaceae are rare. *Viola baoshanensis* is an amazing Cd hyperaccumulator in metalliferous areas of China, whereas its relative *V. inconspicua* is a non-tolerant accumulator that resides at non-metalliferous sites. Here, comparative studies by transcriptome sequencing were performed to investigate the key pathways that are potentially responsible for the differential levels of Cd tolerance between these two *Viola* species. A cascade of genes involved in the ubiquitin proteosome system (UPS) pathway were observed to have constitutively higher transcription levels and more activation in response to Cd exposure in *V. baoshanensis*, implying that the enhanced degradation of misfolded proteins may lead to high resistance against Cd in this hyperaccumulator. Many genes related to sucrose metabolism, especially those involved in callose and trehalose biosynthesis, are among the most differentially expressed genes between the two *Viola* species, suggesting a crucial role of sucrose metabolism not only in cell wall modification through carbon supply but also in the antioxidant system as signaling molecules or antioxidants. A comparison among transcriptional patterns of some known transporters revealed that several tonoplast transporters are up-regulated in *V. baoshanensis* under Cd stress, suggesting more efficient compartmentalization of Cd in the vacuoles. Taken together, our findings provide valuable insight into Cd hypertolerance in *V. baoshanensis*, and the corresponding molecular mechanisms will be useful for future genetic engineering in phytoremediation.

## 1. Introduction

Cadmium (Cd) is a non-essential trace element that can cause toxic reactions in plants and can be easily transferred into vegetative cover and ultimately enter the food chain, becoming a threat to human health [1]. Therefore, Cd is considered a primary cause of soil pollution, and the control of risks related to Cd exposure has become a worldwide concern. The development of efficient phytoremediation and strategies to reduce Cd concentrations in crops are the two most promising strategies for preventing health risks from Cd contamination [2,3]. Indeed, both strategies require deep understanding of the molecular mechanisms involved in Cd absorbance, internal translocation and detoxification. A class of rare plants called hyperaccumulators, which possess extremely high tolerance and high accumulation of heavy metals, have recently evoked considerable interest as model plants for studying plant responses to heavy metal stress, and they are potential genetic resources for the development of future genetic engineering technologies [4].

To date, there have been reports of over 500 hyperaccumulators, while only a small fraction of them have been recognized as Cd hyperaccumulators with the distribution being among the Brassicaceae, Crassulaceae and Violaceae families [5,6,7,8]. Several basic physiological processes, possibly shared by most hyperaccumulators, have already been proposed for the Cd hyperaccumulation and hypertolerance, such as transporter-mediated absorbance and root-to-shoot transportation [9], sequestration of Cd chelates and Cd^2+^ to the vacuole or cell wall [10,11], and scavenging of reactive oxygen species (ROS) [12]. The critical role of the *HMA4* gene (encoding a heavy metal ATPase) in enhanced root-to-shoot translocation within the xylem has a particularly interesting cause, which is likely a highly conserved mechanism in different hyperaccumulators across several phyla [13]. However, most current molecular biological studies focus on the most well-known Cd hyperaccumulators *Arabidopsis halleri* and *Noccaea caerulescens* from the family Brassicaceae by taking advantage of the genetic resources developed for *A. thaliana* [14]. It is generally accepted that most of these hyperaccumulators have independent origins [15], indicating the existence of different genetic architectures for hyperaccumulation traits among these various hyperaccumulators. Therefore, expanding the scope of taxa may provide new insights into the molecular mechanisms of the hyperaccumulation traits [13]. 

Previous studies have suggested that tolerance and accumulation are separated traits that are mediated by genetically and physiologically distinct mechanisms, and thus, the examined plants can be assigned into four categories: tolerant accumulators, non-tolerant accumulators, non-tolerant non-accumulators and tolerant non-accumulators [16]. Although the progression of more detailed transcriptomic descriptions of hyperaccumulators has benefited from next-generation sequencing (NGS) technologies, some notable questions are still present in the majority of comparative analyses between tolerant accumulators and non-tolerant non-accumulators. For example, comparative studies on *A. halleri* versus *A. thaliana* [17], *N. caerulescens* versus *A. thaliana* [14], and *Sedum plumbizincicola* versus *S. alfredii* [18] may have generated confusing results, since they could not clearly distinguish the genes responsible for metal tolerance versus those responsible for metal accumulation. When we compare the tolerant accumulators and non-tolerant accumulators, this misleading information can be avoided by controlling the accumulative traits by setting them as constant variables while focusing on the mechanisms responsible for differential metal tolerance. However, transcriptome-based comparative analyses between tolerant accumulators and non-tolerant accumulators are still lacking.

A large number of hyperaccumulators in Violaceae have been reported in Asian and European countries, for example, *V. baoshanensis* for the hyperaccumulation of Cd [19,20], *V. calaminaria* [20] and *V. lutea* [21] for zinc (Zn), and *Rinorea niccolifera* [22] for nickel (Ni). Previous studies on the responses of *Viola* species to Cd stress were mostly limited to ecological and physiological levels, and only few studies were done at the transcriptional level, while comparative analyses with closely related non-metalliferous species are lacking. *V. baoshanensis*, growing on Baoshan lead/zinc mines in Hunan Province of China was identified by us as both a Cd-hyperaccumulator and a strong accumulator of Pb (lead) and Zn [19,23]; whereas, interestingly, *V. inconspicua* from non-contaminated sites showed less tolerance to Cd than *V. baoshanensis*, but with similar amounts of Cd accumulation in roots and shoots. Accordingly, the striking phenotypic difference between these two *Viola* species provides an exceptional opportunity to elucidate the molecular mechanisms for their differential levels of Cd tolerance on the basis of controlling the Cd accumulation traits. In this study, we sequenced and compared the transcriptomes of *V. baoshanensis* and *V. inconspicua* on an Illumina sequencing platform to investigate the gene transcription patterns of these two distinct *Viola* species under Cd exposure. We attempted to identify candidate genes involved in the Cd detoxification regulatory networks and also tested whether those known mechanisms from Brassicaceae are conserved among Violaceae.

## 2. Results

### 2.1. Metal Accumulation and Tolerance in the Two Viola Species from Hydroponic Experiments

**Figure 1:**
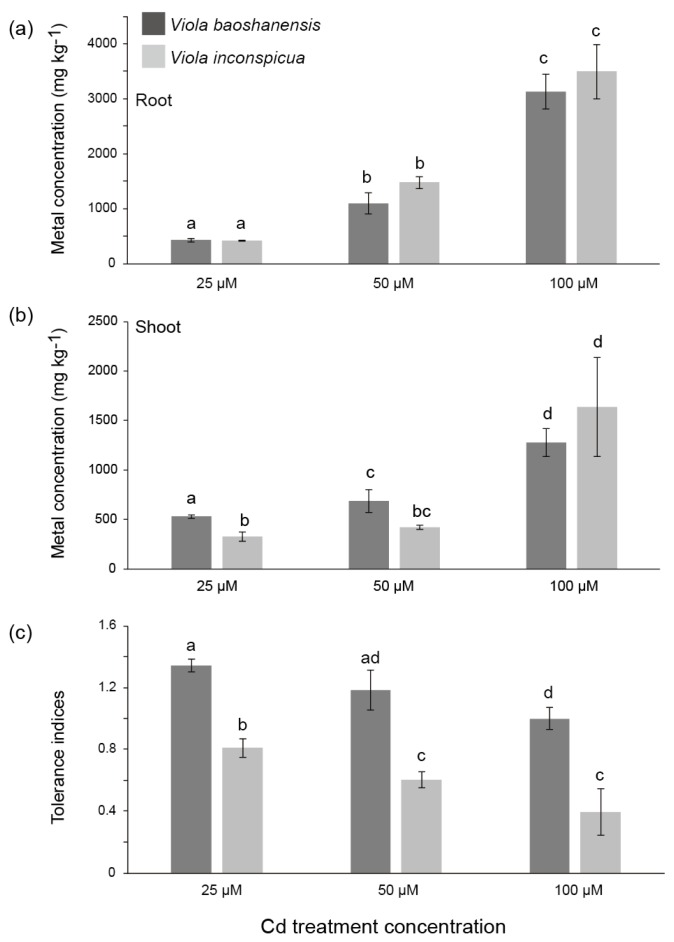
Concentrations of Cd in *Viola baoshanensis* and *V. inconspicua* after different Cd treatments. (**a**) roots, (**b**) shoots, and (**c**) Tolerance indices. The different letters above the columns (*n* = 4), using ANOVA analysis, indicate significant differences (*p* < 0.05) among the Cd treatments.

In order to evaluate the different capabilities for Cd accumulation and tolerance between these two *Viola* species, we performed hydroponic Cd stress experiments. We compared the Cd concentrations in the roots (Figure 1a) and shoots (Figure 1b) and calculated the tolerance indices (ratios of total dry biomass in the Cd treatments and controls) in the roots and shoots of the two Cd-treated *Viola* species (Figure 1c). Finally, we observed that the tolerance indices of *V. baoshanensis* from the contaminated sites were >1 in the 25 and 50 μM Cd treatments, while they were almost equal to 1 in the 100 μM Cd treatments (see Figure 1c); however, all these values were significantly higher than those in *V. inconspicua samples*, which were collected from the non-contaminated sites (see more details in Figure 1c). Interestingly, among the 25, 50 and 100 µM Cd treatments, *V. baoshanensis* took up similar levels of Cd compared with *V. inconspicua* in both the roots (Figure 1a) and the shoots (Figure 1b). In summary, our results indicate that the two *Viola* species exhibit a great difference in Cd tolerance, while they have a similar capacity to accumulate exogenous Cd.

### 2.2. Summary of the Data for Transcriptome Sequencing, De Novo Assembling, and Annotation

To investigate the potential molecular mechanisms of Cd hypertolerance in *V. baoshanensis*, we sequenced and compared the transcriptome data between *V. baoshanensis* and *V. inconspicua* under Cd stressed conditions. The roots and shoots of both *Viola* species were harvested from controls or 100 µM Cd treated groups (with three biological replicates), resulting in a total of 24 cDNA libraries that produced a total of 2500 million paired reads (Table S1). In order to improve the quality of the sequences, we trimmed each read by using quality scores (only those higher than 20 at the 3′-end were kept) and removed those reads with excess non-sequenced (N) bases. Subsequently, these clean reads were used separately for further *de novo* assembling by Trinity and TGICI (see more details in Section 4.5). As a result, a total of 105,280 transcripts (>300 bp) were assembled for *V. baoshanensis* and 101,616 contigs for *V. inconspicua* (before filtering; Table 1). 

After filtering of low coverage and short ORFs (open reading frames), we obtained the final assemblies with 82,854 protein-coding transcripts for *V. baoshanensis* and 80,059 for *V. inconspicua* (Table 1). To assess the completeness of our transcriptome assemblies, we submitted the two final transcript sets to a BUSCO (Benchmarking Universal Single-Copy Orthologs) evaluation [24], which revealed that the majority of the Eudicotyledon core genes had been successfully recovered in the two assemblies (Table S2). These data indicate that our two *Viola* transcriptome assemblies were of high quality.

To compare inter-species differences in transcription level, we identified 19,794 putative orthologous transcripts between *V. baoshanensis* and *V. inconspicua* by the RBH method with an E-value cutoff of 1.0 × 10^−10^ and a coverage threshold of 50% (see more details in Section 4.5). Despite the different quality of the two *Viola* assemblies reflected by their N50 sizes and the BUSCO completeness assessment, the length distribution of orthologous contigs demonstrated that the orthologous transcript sets of *V. baoshanensis* and *V. inconspicua* have similar length distribution (Figure S1b) and similar contig N50 sizes (2223 versus 2067 bp). That is to say, the quality of both orthologous transcript sets is comparable across the two *Viola* species, suggesting the reliability of our interspecies comparison [25].

**Figure 2:**
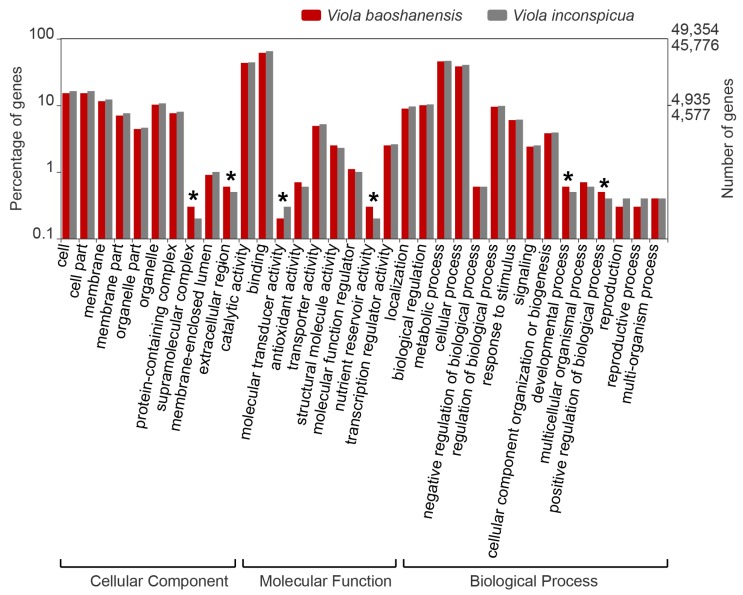
Gene Ontology (GO) distributions for *Viola baoshanensis* (red) and *V. inconspicua* (grey). Annotation results were mapped to categories in the second level of GO terms, respectively. Those GO terms that contain less than 0.1 % of the total genes were excluded from this graph. The asterisks represent significant differences (*p* <0.05) between the two *Viola* species. See more details in Table S14.

The GO (Gene Ontology), KEGG (Kyoto Encyclopedia of Genes and Genomes), and InterproScan databases were employed to annotate the two transcript sets (Table 1). A total of 49,354 (46.8%) genes were mapped to 129,359 GO terms, and 45,776 (45.1%) genes were mapped to 128,847 GO terms for *V. baoshanensis* and *V. inconspicua*, respectively. Detailed proportions of the GO annotation for individual assembly are shown in Figure 2, indicating that molecular functions, biological processes, and cellular components were well represented. By the way, we observed a high GO distribution similarity between these two *Viola* species (see Figure 2). 

In the cellular component category, cell part (15.2% versus 16.3%) and membrane-related functions (11.5% versus 12.3%) were the most abundant. The highest proportions of the mapped GO terms for “Molecular Function” for the two *Viola* species were related to binning (61.2% versus 64.9%) and catalytic activity (43.3% versus 44.1%). Under the “Biological Process”, metabolic process and cellular process were the most enriched terms, comprising 22,446 (45.5%) and 18,950 (38.4 %) genes in *V. baoshanensis* and 21,267 (46.5%) and 18,493 (40.4%) genes in *V. inconspicua*, respectively. We also found that several GO terms significantly differed between these two *Viola* species. For example, under the “Cellular Component” category, *V. baoshanensis* had a higher percentage of genes in the extracellular region and the supramolecular complex (*p* = 0.019) when compared with *V. inconspicua*. In the “Molecular Function” category, we observed that genes in *V. baoshanensis* were significantly over-represented in terms of nutrient reservoir activity (*p* = 0.003), of which seed storage proteins were the main component, the seed storage proteins accumulate significantly in the developing seed, whose main function is to act as a storage reserve for nitrogen, carbon, and sulfur. while molecular transducer activity was less represented (*p =* 0.008) in *V. baoshanensis*. In the last major category, *V. baoshanensis* had more genes related to developmental process (*p* = 0.004) but less genes related to positive regulation of biological process (*p* = 0.004).

### 2.3. Analyses of Differential Expression (DE) and Functional Enrichment

After quantification of transcripts with RNA-Seq by RSEM (see more details in Section 4.6), we employed edgeR to compare the transcriptional changes between *V. baoshanensis* and *V. inconspicua* in response to Cd and also identified the differentially expressed genes (DEGs) in the two species to gain a comprehensive insight into the molecular mechanisms underlying the differences in Cd tolerance.

**Figure 3:**
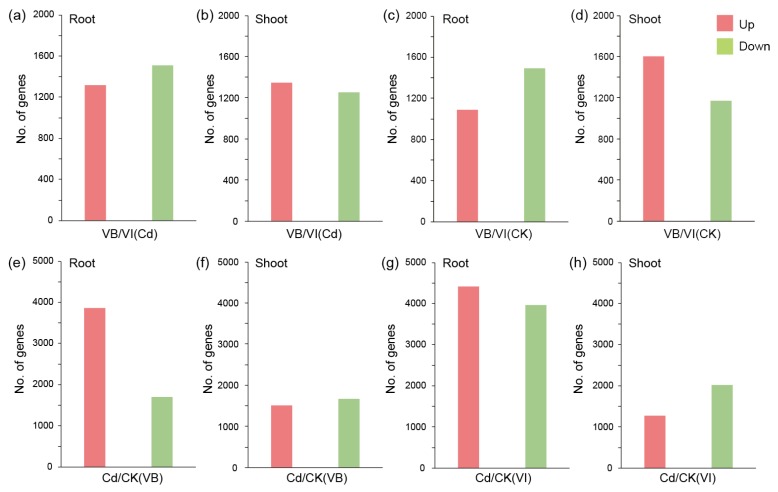
Differentially expressed genes (DEGs) between Cd-treated and control samples in both *Viola* species. Numbers of the genes with significantly different transcription levels (up- or downregulated) between two species in roots (**a**) and shoots (**b**) under the Cd stressed condition, or in the roots (**c**) and shoots (**d**) of the control samples, were summarized for comparison. DEGs between the Cd treated and control samples in VB roots (**e**), VB shoots (**f**), VI roots (**g**), and VI shoots (**h**). CK, control; VB, *Viola baoshanensis*; VI, *Viola inconspicua*.

To determine the genes that responded to the Cd treatment in both *Viola* species, mRNA profiles of Cd treated and untreated (control) roots and shoots were compared (Figure 3 and Figure S2). In *V. baoshanensis*, a total of 5564 transcripts were determined to be DEGs (with *q* values < 0.01 and log2 (fold change) > 2) between the Cd treatment and the control in roots, in which 3,867 were upregulated and 1697 were downregulated in response to the Cd treatment (Figure 3e). A total of 3192 DEGs were determined in the shoots; among them, 1514 were upregulated and 1678 were downregulated respectively in response to the Cd treatment (Figure 3f). However, in *V. inconspicua*, a total of 8380 and 3299 differentially expressed transcripts were identified in the roots (4410 upregulated and 3970 downregulated; Figure 3g) and the shoots (1275 upregulated and 2024 downregulated; Figure 3h) in response to the Cd treatment. A Venn diagram was used to show the specific upregulated transcripts in *V. baoshanensis* (Figure S3). In total, 570 and 403 transcripts in the orthologous gene sets were induced differently in response to Cd between the two *Viola* species in roots and shoots, respectively (see detailed information in Tables S9 and S10). It seems that the two *Viola* species have more divergent responses in the shoot compared with the root under Cd stress, due to a noticeably smaller fraction of commonly induced transcripts in shoots than roots (9.97% versus 36.7%).

For the interspecies comparisons, read counting and DE analysis were restricted to the orthologous genes annotated in *V. baoshanensis* and *V. inconspicua*, which were identified by using the reciprocal best blast hits method. After the DE analysis, we identified 2823 and 2602 DEGs between the two species in the roots (Figure 3a,c) and the shoots (Figure 3b,d), respectively. In the Cd treated roots, 1316 and 1507 genes were upregulated in *V. baoshanensis* (Figure 3a) and *V. inconspicua* (Figure 3a), respectively. Within the Cd treated shoots, 1347 and 1255 genes were upregulated in *V. baoshanensis* (Figure 3b) and *V. inconspicua* (Figure 3b), respectively. 

To determine the functional significance of these interspecies and intraspecies variances in response to Cd treatments, we implemented GO classifications and enrichment analysis for these DEGs (for more information see Figures S4 and S5). A similar pattern was observed between *V. baoshanensis* and *V. inconspicua* in response to Cd exposure (Figure S5); the overrepresented GO terms of the two phenotypes presented a significant overlap for shoots (Figure S5c,d), and the genes related to antioxidant metabolism (such as reactive oxygen species metabolic process and antibiotic metabolic process) were enriched in both species (Figure S5c). However, genes related to metal internal translocation (such as divalent metal ion transport, inorganic ion transmembrane transport, metal ion transmembrane transporter activity, and divalent inorganic cation transmembrane transporter activity) were enriched specifically in *V. baoshanensis* (Figure S5c,d). 

For the roots in *V. baoshanensis*, the most representative categories were regulation of cellular biosynthetic processes, sulfur metabolism-related GO terms (including sulfate transport and sulfate reduction) and ubiquitin-related GO terms (such as ubiquitin-protein transferase activity and ubiquitin-like protein transferase activity; Figure S5b). However, we noted that the ubiquitin related GO terms is not enriched in *V. inconspicua* roots (Figure S5b), suggesting that these genes may be related to the differential Cd tolerance between the two *Viola* species. 

Interestingly, for the interspecies comparisons (Figure S4), we observed that DEGs with higher transcription levels in *V. baoshanensis* roots than in *V. inconspicua* roots were overrepresented by oxidation–reduction processes, tetrapyrrole binding, and transition metal ion transmembrane transporter activity (Figure S4c). In *V. baoshanensis* shoots, however, upregulated DEGs were enriched by microtubule motor activity, heme binding, antioxidant activity, and structural constituents of the cell wall (Figure S4d).

### 2.4. Ubiquitin Proteosome System (UPS) Pathway-Related Genes: Response to Cd Stress

**Figure 4:**
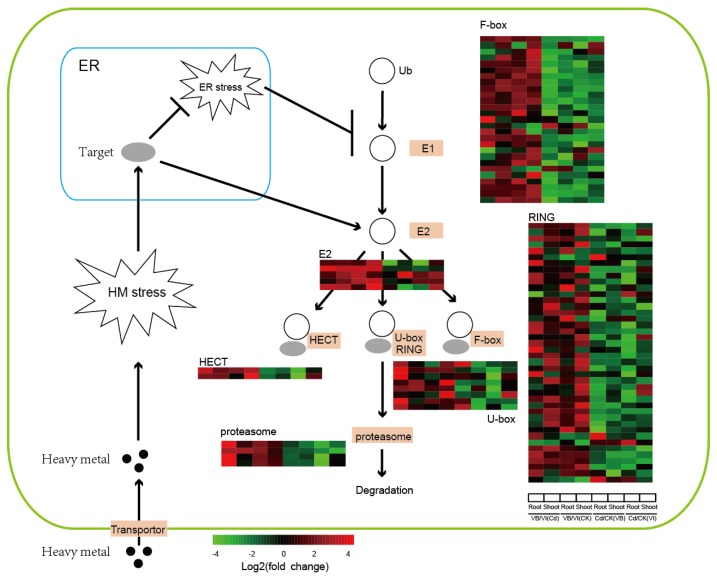
DEGs involved in the ubiquitin proteasome system (UPS) pathway with constitutively higher transcription levels in *Viola baoshanensis*. The heat maps for the relative transcription of DEGs were calculated by log2-fold changes. VB/VI(Cd), VB/VI(CK), Cd/CK(VB), and Cd/CK(VI) represent ratios of transcription levels between *V. baoshanensis* (VB) and *V. inconspicua* (VI) in the Cd treatment (Cd) and control (CK), and ratios of transcription levels of Cd/CK in VB and VI, respectively. E1, Ubiquitin-activating enzymes; E2, ubiquitin conjugases; HECT, U-box, F-box and RING, subfamilies of ubiquitin ligases. See more details about activation of the UPS pathway by ER stress in Table S11, Section 2.4 and Section 3.1 (under the Discussion).

The GO enrichment analysis showed that ubiquitin-protein transferase activity was specifically and significantly enriched in *V. baoshanensis* (Figure S5b), suggesting that the UPS pathway (Figure 4) may play important roles in the differential Cd tolerance between the two *Viola* species. These DEGs in the UPS pathway were further compared between the two phenotypes (Figure 4; Tables S3, S4 and S11). Interestingly, we identified differential expression of transcripts at almost all steps of this pathway (Figure 4), and *V. baoshanensis* apparently has more upregulated genes than *V. inconspicua* for responding to the Cd stress (Tables S3 and S4). Totals of 125 and 36 UPS-related genes were upregulated by Cd in *V. baoshanensis* roots and shoots, respectively; however, fewer upregulated DEGs were observed in *V. inconspicua* roots and shoots, especially regarding the Fbox and Ubox E3 ligase gene families (see more details in Table S11). 

For the interspecies comparisons, we observed that 88 UPS-related genes had significantly higher transcription levels in *V. baoshanensis* compared with *V. inconspicua*, including 5 in the E2 gene family, 27 in the Fbox gene family, 2 in the HECT gene family, 42 in the RING gene family, 8 in the Ubox gene family, and 4 in the proteasome gene family (Tables S3 and S11). Interestingly, significantly fewer UPS-related genes were transcribed at significantly higher rates in *V. inconspicua* than in *V. baoshanensis* (Table S4).

### 2.5. DEGs Involved in Sucrose Metabolism

**Figure 5:**
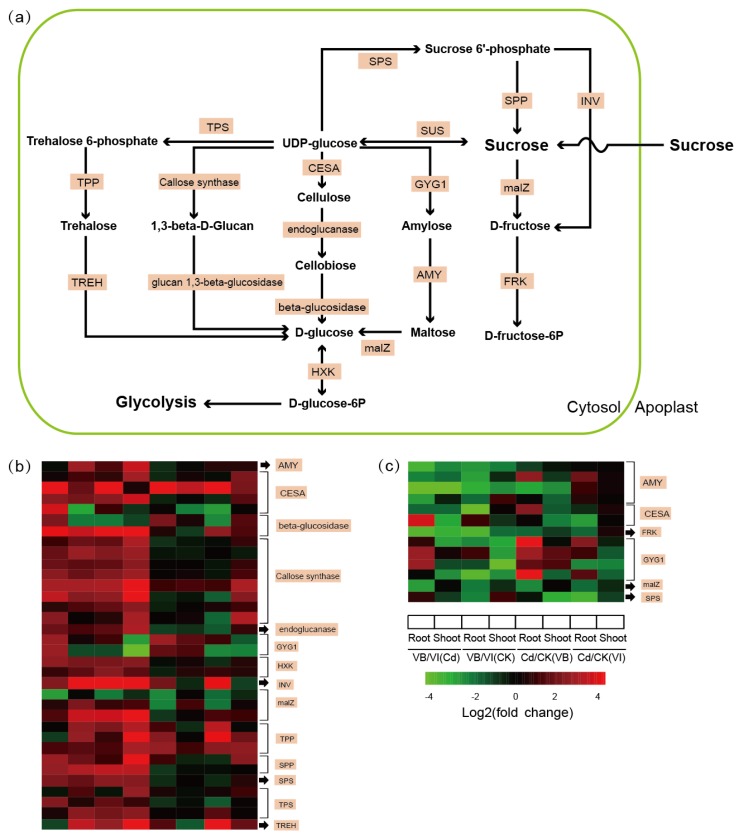
DEGs involved in sucrose metabolism with differential transcription levels in the two *Viola* species. (**a**) Schematic diagram of the sucrose metabolism pathway in *Viola*. (**b**) Heat maps of genes involved in sucrose metabolism with constitutively higher transcription levels in *Viola baoshanensis*. (**c**) Heat maps of genes with constitutively higher transcription levels in *V. inconspicua*. AMY, alpha-amylase; CESA, cellulose synthase; FRK, Fructokinase; HXK, Hexokinase; GYG1, glycogenin; INV, beta-fructofuranosidase; malZ, alpha-glucosidase; SPP, sucrose-6-phosphatase; SPS, sucrose-phosphate synthase; TPP, trehalose 6-phosphate phosphatase; TPS, trehalose 6-phosphate synthase; TREH, alpha trehalase. See more details in Table S12, Section 2.5 and Section 3.2.

Our KEGG enrichment analysis demonstrated that the constitutive DEGs in *V. baoshanensis* were enriched in the sucrose metabolism pathway (Figure 5a) in both roots and shoots (Figures S6 and S7), implying that this important pathway may be responsible in part for the phenotypic difference between the two *Viola* species. For the interspecies comparisons, we found that a total of 40 genes, encoding 19 enzymes in the starch and sucrose metabolism pathway, presented higher transcription levels in *V. baoshanensis* than *V. inconspicua* (see more details in Tables S5 and S6). 

Nine genes encode 1,3-beta-glucan synthases (key enzymes for callose biosynthesis), and none of the members of this gene family had a higher transcription level in *V. inconspicua* (Figure 5; Tables S5 and S6). For a comparison in the Cd treated samples and controls, many genes were upregulated in response to Cd stress in the roots (41 versus 50) and shoots (22 versus 33) of *V. baoshanensis* and *V. inconspicua*, respectively (see more details in Table S12), suggesting a common activation of the sucrose metabolism pathway by Cd in both *Viola* species. 

### 2.6. DEGs Encoding Metal Transporter Proteins

**Figure 6:**
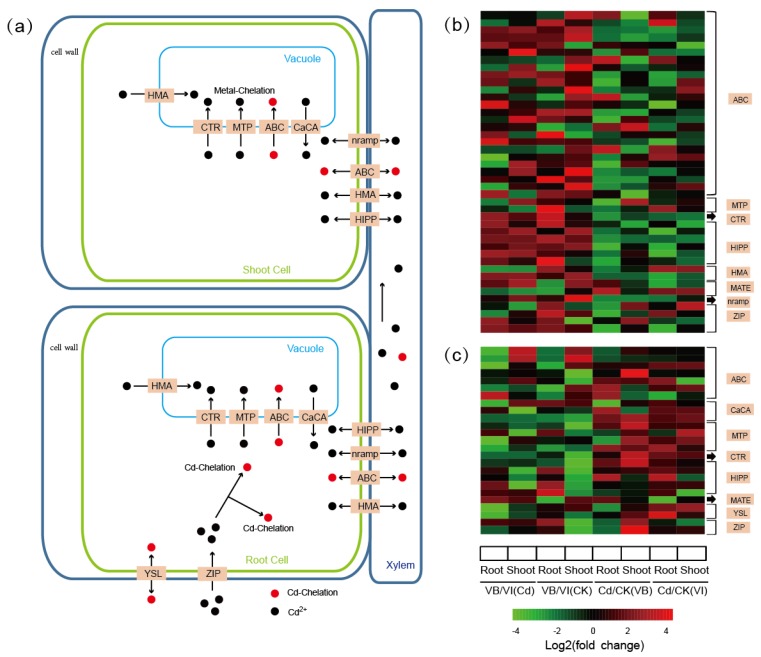
DEGs encoding putative metal transporters. (**a**) A schematic representation of the main processes for differential Cd uptake and internal translocation in *Viola* species. (**b**) Heat maps of genes encoding putative metal transporters with constitutively higher transcription levels in *V. baoshanensis*. (**c**) Heat maps of genes encoding putative metal transporters with constitutively higher transcription levels in *V. inconspicua*. ABC, ATP-binding cassette transporter; CaCA, the Ca^2+^: cation antiporter protein; CTR, the copper transporter protein; HIPP, heavy metal-associated isoprenylated plant protein; HMA, heavy metal ATPase; MATE, multi-antimicrobial extrusion protein; MTP, metal tolerance protein; Nramp, natural resistance-associated macrophage protein; YSL, yellow stripe-like protein; ZIP, the Zinc/Iron permease protein. See more details in Table S13, Section 2.6 and Section 3.3.

Metal transporters have been widely regarded as the critical components in hyperaccumulators due to their Cd accumulation, root-to-shoot transportation and internal sequestration [26]. Among the known 10 transporter families, 43 transcripts (24 ABCs, 2 MTPs, 1 CTR, 6 HIPPs, 2 HMAs, and 1 MATE, 1 NRAMP, and 5 ZIPs; see Figure 6) were identified as orthologous DEGs with constitutively higher transcription levels in *V. baoshanensis*; meanwhile, less transcripts from these transporter families (especially in ZIPs, ABCs, and HMAs) showed constitutively higher transcription levels in *V. inconspicua* (Tables S7 and S8).

Within the 24 ABC DEGs, 12, 6, 5, and 1 belong to the ABCG, ABCC, ABCB, and ABCA subfamilies respectively. Two HMA genes, *HMA4* and *HMA5*, were shown to be transcribed at higher levels in *V. baoshanensis*; in particular, the *HMA4* gene presented a ~100-fold higher level in *V. baoshanensis* than in *V. inconspicua* in both the Cd treated and control samples (Figure 6b; Table S13). For the comparisons between the Cd treated and control samples, 14 genes encoding ZIP proteins were upregulated in *V. baoshanensis* shoots in response to the Cd stress, while only 2 were upregulated in *V. inconspicua* shoots (Figure 6b; Table S13). On the other hand, we also found that several genes related to the YSL family and CaCA family were more activated in *V. inconspicua* (Figure 6c; Table S13). Our findings may reveal the distinct strategies in *V. baoshanensis* and *V. inconspicua* for Cd transportation and sequestration as a consequence of different surroundings.

## 3. Discussion

Despite the consecutive reporting of hyperaccumulators in Violaceae, progress in understanding the hypertolerance abilities of metallicolous plants in this class has been limited to metal mobilizing in cells at a physiological level [21], while transcriptome-level studies are rare. Here, we compared the transcriptomes of two *Viola* species, a tolerant hyperaccumulator *V. baoshanensis* and a non-tolerant accumulator *V. inconspicua*, from metalliferous and nonmetalliferous sites respectively. Several key pathways are proposed as being associated with their differential Cd tolerance.

### 3.1. How Does the UPS Pathway Enhance Heavy-metal Resistance in Plants?

As shown in Figure 3, a cascade of genes involved in the UPS pathway displayed constitutively higher transcription levels and greater activation in response to Cd stress in *V. baoshanensis* than in *V. inconspicua*. In plants, the UPS pathway acts through the sequential actions of a cascade of enzymes (see more details in Figure 4) to add multiple copies of the protein ubiquitin (ub) to a substrate protein that is then targeted for degradation by the 26S proteasome [27]. Both transcriptome and proteome studies in various plant species have shown that UPS pathway related genes can be activated by heavy metals, such as Cd, Cu (copper), Hg (mercury), and Pb. For instance, the ubiquitin-dependent proteolysis pathway in yeast was activated in response to Cd exposure [28], and the polyubiquitin genes in common bean and rice were strongly stimulated by Hg [29] and low concentrations of Cd [30] respectively. 

However, there are two distinct theories to explain how the overexpression of genes in the UPS cascade enhances the tolerance of heavy metals. The first one focuses on the UPS pathway, since it is a rapid and effective method for precise degradation of misfolded proteins that are induced by heavy metal ions [31,32,33]. Previous studies demonstrated that heavy metal ions inhibit the refolding of chemically denatured proteins in vitro, obstruct protein folding in vivo, and stimulate the aggregation of nascent proteins in living cells [34]. Together with evidences that yeast mutants in the proteasome are hypersensitive to Cd, it was suggested that heavy metal tolerance can be mediated by degradation of abnormal proteins [28]. Another theory depends on the ubiquitination process, which may indirectly mediate the tolerance of heavy metals by the regulation of heavy metal transporters [35,36,37]. For example, rice *OsHIR1* E3 ligase protein is able to control metal uptake through regulation of the *OsTIP4-1* protein via ubiquitination [36]. In the present study, for the first time, we found that the UPS pathway related genes have constitutively higher transcription levels in tolerant hyperaccumulators than closely related non-tolerant accumulator species in both roots and shoots; however, it is hard to infer which theory or whether both of them may mediate the Cd hypertolerance in *V. baoshanensis*. The detailed mechanisms are worthy of in-depth investigation. Moreover, it seems that the effects of the UPS pathway on heavy metal stress may not be generalized, since the UPS system can be applied to develop biotechnological tools not only to reduce metal concentrations for food safety but also to strengthen metal accumulations for phytoremediation [37].

### 3.2. Relations of Sucrose Metabolism with Heavy-Metal Stress in Plants

Sucrose and starch metabolism play pivotal roles in development, the stress response and synthesis of essential components (including proteins, cellulose, and starch) in higher plants [38]. For example, the addition of dialdehyde starch derivatives in heavy metal-contaminated soils limited the negative impact of these metals both in terms of yield and heavy metal content in maize [39]. In this study, we observed a significant difference in transcription levels at almost all steps of sucrose and starch metabolism (Figure 5a) between *V. baoshanensis* and *V. inconspicua* (Figure 5b). These data are consistent with previous descriptions of the transcriptome in response to heavy metal stress in other phyla [40,41,42]; however, detailed descriptions and discussion of this pathway are still limited. 

Hexokinases (HXKs) and fructokinases (FRKs) are two families of enzymes with the capacity to catalyze the essential irreversible phosphorylation of glucose and fructose, and therefore, they may play central roles in the regulation of plant sugar metabolism. It has been suggested that in vivo, HXKs probably mainly phosphorylate glucose, whereas fructose is phosphorylated primarily by FRKs [43]. Interestingly, 2 HXKs homologous to *Arabidopsis HXK1* were found to have constitutively higher transcription levels in *V. baoshanensis* than in *V. inconspicua*, while 2 FRKs homologous to *Arabidopsis FRK1* and *FRK2* were constitutively higher in *V. inconspicua*. A previous study has shown that the reduction of *FRK2* activity in aspen (*Populus tremula*) led to thinner fiber cell walls with a reduction in the proportion of cellulose by decreased carbon flux to cell wall polysaccharide precursors [44], indicating that the cell wall biosynthesis may be repressed in *V. baoshanensis* by low expression of FRK and thus may reduce the capacity of cell walls to act as barriers against Cd translocation. HXKs have been demonstrated to play potential roles in the uptake of Zn in roots, since the HXK-dependent transporter *ZIP11* is unrelated to sugar sensing but may be related to sugar metabolism downstream of HXK [45]. These findings are in accordance with our present observation that *ZIP11* transcribed with significantly higher levels in *V. baoshanensis* roots than in *V. inconspicua* roots under Cd stress. 

Uridine diphosphate glucose (UDP-Glc), one product of the sucrose cleavage reactions, is the substrate for biosynthesis of callose, which is associated with the plasma membrane. In this study, we found that *V. baoshanensis* had constitutively higher transcription levels of two related enzymes, callose synthases (β-1,3-glucan synthases) and β-1,3-glucanases, which can produce and break down callose respectively [46]. Of the 8 upregulated genes encoding callose synthases and the 2 genes encoding glucan 1,3-beta-glucosidase in *V. baoshanensis*, 7 CALSs (2 *CALS10*, 2 *CALS7*, *CALS5*, *CAL3,* and *CLA9*) and 2 *pdBG1s* have been reported to localize in the plasmodesmata and to regulate callose and plasmodesmatal permeability [47,48,49], suggesting an alteration of the plasmodesmatal permeability between the two *Viola* species. With the evidence that accumulation of callose accompanies a reduction in plasmodesmatal permeability leading to reduced growth and depletion of the stem cell population [50], the decreased callose levels in response to heavy-metal stress may buffer the negative effects on primary root growth, and thus to increase heavy metal trafficking through the plasmodesamata [51] and subsequently, increase resistance to heavy metals [52]. Hence, we speculate that *V. baoshanensis* may have decreased the plasmodesmatal permeability to a greater extent than *V. inconspicua*, thereby enhancing Cd tolerance. 

In addition to cell wall modification, which is regulated by sucrose metabolism through the carbon supply, sugars also play protective functions against various abiotic stresses in several physiological processes by acting as signaling molecules in plants [53,54]. Six genes, encoding trehalose-6-phosphate phosphatase (TPP) and trehalose-6-phosphate synthase (TPS), which are involved in trehalose biosynthesis in the sucrose metabolism pathway, were found to have significantly elevated transcription levels in *V. baoshanensis*, implying that trehalose-6-phosphate (T6P) and trehalose may play significant roles in the phenotypic difference in Cd tolerance between the two *Viola* species. Several previous studies support this hypothesis. For instance, enhanced endogenous trehalose levels in rice seedlings significantly mitigated the toxic effects of excessive Cu^2+^ by inhibiting Cu uptake and regulating the antioxidant and glyoxalase systems [55]. Another case is that overexpression of *Arabidopsis* trehalose-6-phosphate synthase in tobacco plants (*AtTPS1*) was shown to lead to better acclimation to Cd and excess Cu than in the wild-type [56]. Regarding the mechanisms involved in the use of trehalose biosynthesis to mediate responses to heavy metals, the production of antioxidants or antioxidant enzymes to alleviate excessive heavy metals has been proven to be induced by trehalose-6-phosphate [53,55,56,57]. In line with our expectations, several genes with significantly higher transcription levels in *V. baoshanensis* were enriched in the GO term of peroxidase activity. Moreover, trehalose can act directly as an antioxidant on excess ROS [58]. Interestingly, the effects of trehalose on heavy metal uptake seem to be contradictory. Reduced Cu uptake was reported in rice seedlings with trehalose treatment [55], while higher transcription levels of *AtTPS1* in tobacco resulted in more Cd and Cu accumulation than in a transgenic line [56].

### 3.3. Contributions of Transporter Proteins to Heavy Metal Tolerance in Plants

It is well known that the enhancement of transporters for essential elements (such as Fe^2+^, Zn^2+^, and Ca^2+^) may be involved in non-essential metal uptake and transport in hyperaccumulators [59]. To test whether these mechanisms of heavy metal hyperaccumulation are conserved in the *Viola* phyla, we identified and compared several related gene families between the two *Viola* transcriptome sets. ZIP family genes are the most important Zn/Fe plasma membrane transporters, and in this study 5 ZIP transporters (*ZIP1*, *3*, *4*, *5,* and *11*) displayed higher levels of transcription in *V. baoshanensis* than in *V. inconspicua*. Previous studies have shown that *ZIP4* is necessary for the enhanced accumulation of metal ions, and the metal accumulating capacity correlates with higher expression levels of ZIP4 in a known hyperaccumulator *N. caerulescens* [60].

P1B-type ATPases (heavy metal transporting ATPases, HMAs) have been shown to be involved in root-to-shoot long-distance transportation of heavy metals. In particular, *HMA4* is responsible for efficient xylem loading of Cd, and it has been regarded as a key gene for Zn/Cd accumulation in shoots by overexpression in a hyperaccumulator [61]. We found that transcription levels of the *HMA4* gene in *V. baoshanensis* can reach ~100 fold higher than in *V. inconspicua*, although both *Viola* species can accumulate high levels of Cd in the aboveground parts. Thus, we propose that the *HMA4* protein may have other functions besides its role in root-to-shoot metal transportation.

We also observed a member of the Nramp family (*Nramp1*) with constitutively higher transcription levels in *V. baoshanensis*, which was reported to be involved in the influx of Cd across endodermal plasma membrane for root-to-shoot transportation [62]. Although *V. baoshanensis* and *V. inconspicua* have similar abilities to accumulate Cd, we still observed contrasting transcriptional patterns of transporters in relation to metal uptake and root-to-shoot transport, suggesting that the movement of metals from roots to shoots in the two *Viola* species may be regulated by different pathways.

Different from the transporters responsible for heavy metal uptake and root-to-shoot transport, tonoplast transporters usually sequester heavy metals in leaf cellular vacuoles with a central role in the heavy metal homeostasis of plants [63]. Several vacuolar transporters, such as *MTP3* and *COPT5* (using divalent heavy-metal irons as substrates) and 25 ABC transporters (using the heavy metal–phytochelatin (HM-PC) complex as the substrate) were found to have higher transcription levels in *V. baoshanensis* than in *V. inconspicua*, implying that the enhancement of vacuolar compartmenta-lization in *V. baoshanensis* may have contributed to its higher Cd tolerance than *V. inconspicua*.

However, our comparative transcriptome analysis showed constitutively higher transcription levels and more activation of the CaCA gene family and YSL gene family (8 genes homologs to *AtYSL3* and 2 genes homologs to *AtYSL1*), suggesting that they were induced by Cd stress in *V. inconspicua*, especially in the roots. The *YSL* gene family encodes plasma-localized transporters to deliver various heavy metal–nicotianamine (HM–NA) complexes containing Fe(II), Cu, Zn, and Cd, and *YSL3* has been received extensive attention as it may be responsible for internal metal transport in hyperaccumulators in response to metal stress. Nevertheless, the function of *YSL3* may not be conserved across various phyla. Overexpression of *TcYSL3* and *SnYSL3* from the hyperaccumulators *N. caerulescens* and *Solanum nigrum* has been reported to function in metal hyperaccumulation in shoots [41,64], while high expression of *AhYSL3.1* from peanut and rice plants with excess Cu, resulted in a low concentration of Cu in young leaves [65], implying a potential capacity of *YSL3* to reduce metal toxicity by metal efflux. The contradictory expression patterns of HM-NA transporter YSL genes and HM-PC transporter ABC genes in the two *Viola* species may represent two distinct evolutionary lines. We suggest that high expression of MTP, CTR, and ABC genes may enhance vacuolar mobilization in both root and shoot cells to support *V. baoshanensis* with a greater Cd tolerance.

## 4. Materials and Methods

### 4.1. Collection of Plant Samples

Two *Viola* species were investigated in this study. *V. baoshanensis* was collected from anthropogenically contaminated soils from local Baoshan Pb/Zn mines in Guiyang City, Hunan Provinces China, whereas *V. inconspicua* was collected from non-metalliferous sites in Guangzhou City, Guangdong Provinces China.

### 4.2. Hydroponic Experiments

**Figure 7:**
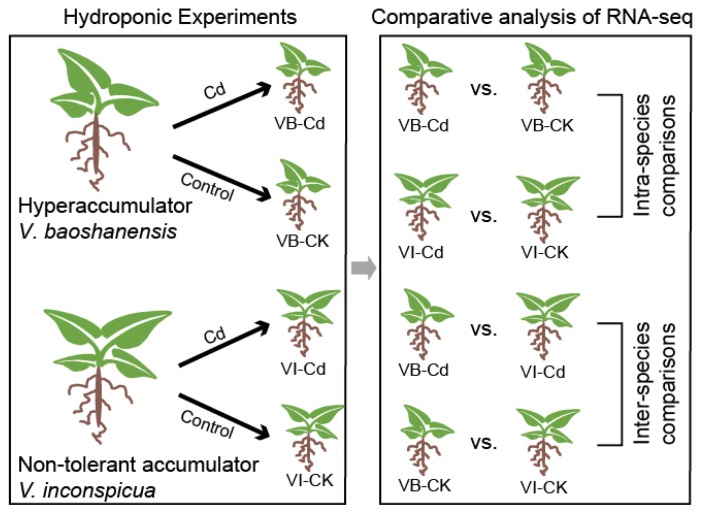
A schematic view of the experimental design for subsequent RNA-seq. VB, *V. baoshanensis*; VI, *V. inconspicua*; CK, control samples.

For hydroponic experiments, tissue-cultured seedlings of *V. baoshanensis* were prepared as described in our previous report [66]. *V. inconspicua* seedlings were collected from natural soils at Sun Yat-Sen University (Guangzhou, China). These seedlings were subsequently cultured in 0.1-strength Hoagland solutions [67]. After 4 weeks of growth, various amounts of Cd were added into the culturing solutions, which were refreshed at 2-day intervals. In the Cd treatments, Cd was supplied as CdCl_2_ at concentrations of 25, 50, or 100 µM; meanwhile, the treatment without Cd addition was regarded as the control (CK). The hydroponic experiments were conducted at 25 °C with an illumination of LD 16/8 in a glasshouse. The root and shoot samples for transcriptome sequencing (RNA-seq, 100 µM Cd) and the Cd elemental analysis were harvested after one month of the Cd exposure tests. A schematic diagram of the experimental design is summarized in Figure 7. The Cd tolerance indices of *V. baoshanensis* and *V. inconspicua* were calculated as the ratios of the total dray biomass in the Cd treatment and the control.

### 4.3. Elemental Analysis of the Hydroponic Samples

The plants collected from the hydroponic experiments were digested with a mixture of concentrated HNO_3_ and HClO_4_ at 5:1 (*v*/*v*) [68]. The concentrations of Cd in plant samples were measured by an Inductively Coupled Plasma Optical Emission Spectroscopy (ICP-OES) (PerkinElmer, Shelton, CT, USA). Quality control was performed using standard reference materials (GBW 07604) purchased from the China Standard Materials Research Center and including blanks in the digestion batches. The recovery rates for all samples were around 90 ± 10%.

### 4.4. RNA Extraction and cDNA Library Preparation

Total RNA was isolated using a HiPure Plant RNA mini kit (Magen, Guangzhou, China). The concentrations and integrity of the isolated RNA samples were evaluated by 1.2% agarose gel electrophoresis and ultraviolet spectrophotometry before cDNA synthesis. The poly(A) RNA was enriched from 2 μg of total RNA using magnetic oligo (dT) beads. The harvested poly(A) RNA samples were fragmented into small pieces using divalent cations under 94 °C for 8 min. cDNA synthesis was performed using the Illumina TruSeq RNA Sample Preparation v2 Kit (Illumina Inc, San Diego, CA, USA). The cleaved RNA fragments were converted into first-strand cDNA using reverse transcriptase and random primers. Second-strand cDNA synthesis was conducted using DNA Polymerase I and RNase H. The synthesized short cDNA fragments were further processed by end repair, adapter ligation, and agarose gel separation. Finally, the correct-sized fragments were selected as templates for PCR amplification with the sequencing primer pairs.

### 4.5. Transcriptome Sequencing, Assembling, and Annotation

Paired-end sequencing (2 × 150 bp) was performed on the Illumina HiSeq2000 sequencing platform at a sequencing depth of 30–50 million reads per library. Preprocessing of those raw short reads by custom script for quality control included the following three steps: (1) elimination of reads with adapter contamination; (2) removal of reads with excess of non-sequenced bases (N; >5% of each read); (3) trimming of low-quality reads (quality value < 20 at the 3′-ends). Quality control of both raw and processed reads was performed with customized Perl scripts. The Transcriptome Shotgun Sequencing project has been deposited at NCBI Sequence Read Archive under the accession number PRJNA524759.

Given the lack of an available reference genome, we subjected the filtered reads to de novo assembling using Trinity [69] with default parameters. Two reference transcriptomes were produced from *V. baoshanensis* and *V. inconspicua*, respectively, by assembling the reads across all tissues (roots and shoots) and the biological replicates obtained from the constructed libraries. To obtain sets of non-redundant transcripts, TGICL-2.1 [70] was employed to reassemble highly similar transcripts with an identity threshold of 0.94. Subsequently, we applied TransDecoder (https://transdecoder. github.io/) to the ORFs for each transcript and removed those transcripts with short ORFs (<100 bp in length) and low transcription (average Transcripts Per Million bases (TPM) < 1). To provide comprehensive descriptions of the final transcript sets, we employed several public databases including Swissprot/Uniprot [71], KEGG (Kyoto Encyclopedia of Genes and Genomes) and InterproScan [72] to annotate these unigenes. We used WEGO [73] to visualize and compare the GO (Gene Ontology) annotation results of *V. baoshanensis* and *V. inconspicua*. Based on the WEGO manual, the Chi-square test of independence was applied to determine whether there were significant differences in the frequencies of genes within GO terms between the two species.

### 4.6. Differential Expression and Statistical Analysis

Paired-end reads from each library were individually mapped to their respective transcriptome assemblies using Bowtie2 [74]. RSEM [75] was used to estimate the transcription and raw counts of each transcript. For intra-species comparisons, all expressed transcripts were used for differential expression analysis between Cd-treated samples and control samples. For interspecies comparisons, in order to directly compare the transcription levels between *V. baoshanensis* and *V. inconspicua*, the differential expression analysis was narrowed down to only those constitutive transcripts that were orthologously presented in the two species. We identified orthologous genes between the two transcriptome assemblies by reciprocal best hits (RBH) BLAST [76]. Differential expression analysis was realized using the Bioconductor package edgeR [77] with parameters (minimum fold change = 4, *p*-value cutoff = 0.01 after FDR correction). The differentially expressed transcripts were then subjected to enrichment analyses, using GOeast [78] for GO enrichment and clusterProfile [79] for KEGG enrichment. We used the entire transcript annotation as the background set when conducting the DEG functional enrichment analysis, which resulted from intraspecies comparisons. For interspecies comparisons, however, we used annotations of orthologous genes as a background set for DEG functional enrichments analysis. The significance was assessed using a hypergeometric test with FDR *p*-value correction (*p* < 0.05). Comparisons of the Cd accumulation in tissues and the tolerance indices between *V. baoshanensis* and *V. inconspicua* were performed using the statistical package SPSS 13.0 for Windows (IBM, Armonk, NY, USA). The data were examined using one-way ANOVA, followed by multiple comparisons using the least significant difference (LSD) test. The level of significance was set at *p* < 0.05 (two-tailed).

## 5. Conclusions

In summary, we sequenced and assembled high-quality transcriptomes for the Cd hyperaccumulator *V. baoshanensis* and its non-tolerant counterpart *V. inconspicua*, with an important contribution to the accumulation of the genetic resources of hyperaccumulators with more diverse taxa. Furthermore, intraspecies and interspecies differential expression genes and related functional enrichments were revealed by comparative analyses. Our results suggest an integrated strategy of Cd detoxification, mediated by UPS-dependent proteolysis, sucrose metabolism and vacuolar mobilization, as being responsible for the Cd hypertolerance of *V. baoshanensis*. The transcriptomic data presented in this study also provide genetic support for deep investigations on the conservation of these candidate genes and pathways in other plants.

## Figures and Tables

**Table 1:** Statistics of the assembling and annotation data for the transcriptomes of two *Viola* species.

Parameter	*Viola baoshanensis*	*Viola inconspicua*
No. of contigs (before filtering)	105,280	101,616
No. of contigs	82,854	80,059
Maximum length of contigs (bp)	67,368	42,149
Average length of contigs (bp)	1984	1348
Contig N50 (bp)	2778	1709
GC content (%)	43.8	42.5
Annotated in KEGG	31,772	31,141
Annotated in GO	49,354	45,646

## Supplementary Materials

Supplementary materials can be found online at https://www.mdpi.com/1422-0067/20/8/1906/s1. Table S1. Statistics of the transcriptome sequencing data. Table S2. Summary of the BUSCO evaluation. Table S3. Statistics of upregulated DEGs in the UPS pathway. Table S4. Statistics of downregulated DEGs in the UPS pathway. Table S5. Statistics of upregulated DEGs in the sucrose metabolism pathway. Table S6. Statistics of downregulated DEGs in the sucrose metabolism pathway. Table S7. Statistics of upregulated DEGs encoding transporters. Table S8. Statistics of downregulated DEGs encoding transporters. Table S9. *V. baoshanensis* specific upregulated transcripts in roots in response to Cd stress. Table S10. *V. baoshanensis* specific upregulated transcripts in shoots in response to Cd stress. Table S11. Fold changes of DEGs involved in the UPS pathway between *V. baoshanesis* and *V. inconspicua*. Table S12. Fold changes of DEGs involved in sucrose metabolism between *V. baoshanesis* and *V. inconspicua*. Table S13. Fold changes of DEGs encoding transporters between *V. baoshanesis* and *V. inconspicua*. Table S14. Detailed proportions of GO terms in *V. baoshanensis* and *V. inconspicua*. Figure S1. Length distribution of entire transcripts and orthologous transcripts in *V. baoshanensis* and *V. inconspicua*. Figure S2. Volcano plots of gene transcription-level changes in the two *Viola* species with different Cd treatments (from 0 µM to 100 µM). Figure S3. A Venn diagram of specific and common up-regulated transcripts in *V. baoshanensis* and *V. inconspicua* in response to Cd stress. Figure S4. Summary of significantly upregulated genes in *V. baoshanensis* compared with *V. inconspicua*. Figure S5. GO enrichment of upregulated DEGs in *V. baoshanensis* and *V. inconspicua*. Figure S6. KEGG enrichment of upregulated DEGs in roots and shoots of *V. baoshanensis* compared with *V. inconspicua*. Figure S7. KEGG enrichment of up-regulated DEGs in *V. baoshanensis* and *V. inconspicua*.

## Abbreviations

ABC	ATP-binding cassette transporters
AMY	alpha-amylase
bglX	beta-glucosidase
BUSCO	Benchmarking Universal Single-Copy Orthologs
Cd	cadmium
CaCA	the Ca^2+^: cation antiporter Family
CESA	cellulose synthase
CTR	the Copper transporter family
DEG	differential expression gene
FRK	fructokinase
GO	Gene Ontology
GYG1	glycogenin
HMA	heavy metal ATPase
HIPP	heavy metal-associated isoprenylated plant protein
HXK	hexokinase
INV	beta-fructofuranosidase
KEGG	Kyoto Encyclopedia of Genes and Genomes
malZ	alpha-glucosidase
MATE	multi-antimicrobial extrusion protein
MTP	metal tolerance protein
Ni	nickel
Nramp	natural resistance-associated macrophage protein
OM	organic matter
Pb	lead
SPP	sucrose-6-phosphatase
SPS	sucrose-phosphate synthase
TPP	trehalose 6-phosphate phosphatase
TPS	trehalose 6-phosphate synthase
TREH	alpha, alpha-trehalase
UPS	ubiquitin proteosome system
YSL	yellow stripe-like family
ZIP	the Zinc/Iron permease family
Zn	zinc
