# Supplementary material for: Comparative Transcriptomic Studies on a Cadmium Hyperaccumulator Viola baoshanensis and Its Non-Tolerant Counterpart V. inconspicua
Source: Int J Mol Sci. 2019 Apr 17;20(8):1906. doi: 10.3390/ijms20081906 (PMC6515270; doi:10.3390/ijms20081906)
Supplement: Supplementary file 1 [file ijms-20-01906-s001.zip › ijms-466616-supplementary-3/Table S1-S8 Figure S1-S7.docx]

**Table S1** Statistics of the transcriptome sequencing data

| **Species** | **Treatments** | | | **Raw data** | | **Clean data** | |
| --- | --- | --- | --- | --- | --- | --- | --- |
|  |  |  |  | **No. of reads** | **Raw reads (Gb)** | **No. of reads** | **Clean reads (Gb)** |
| *V. baoshanensis* | CK | Root | B-1-1 | 103,181,115 | 14.41 | 90,728,222 | 12.25 |
|  |  |  | B-1-2 | 119,219,028 | 16.65 | 104,830,525 | 14.16 |
|  |  |  | B-1-3 | 132,971,345 | 18.58 | 116,923,080 | 15.79 |
|  |  | Shoot | B-2-1 | 122,254,139 | 17.08 | 107,499,329 | 14.52 |
|  |  |  | B-2-2 | 111,968,955 | 15.64 | 98,45,5461 | 13.30 |
|  |  |  | B-2-3 | 102,463,603 | 14.31 | 90,097,306 | 12.17 |
|  | Cd | Root | B-3-1 | 107,034,796 | 14.95 | 94,116,803 | 12.71 |
|  |  |  | B-3-2 | 91,900,510 | 12.84 | 80,809,069 | 10.91 |
|  |  |  | B-3-3 | 78,560,928 | 10.97 | 71,883,249 | 10.04 |
|  |  | Shoot | B-4-1 | 85,460,289 | 11.94 | 78,196,164 | 10.92 |
|  |  |  | B-4-2 | 115,516,409 | 16.13 | 105,697,514 | 14.76 |
|  |  |  | B-4-3 | 66,190,159 | 9.24 | 60,563,995 | 8.46 |
| *V. inconspicua* | CK | Root | C-1-1 | 109,359,973 | 15.28 | 961,613,56 | 12.99 |
|  |  |  | C-1-2 | 112,175,282 | 15.67 | 986,36,886 | 13.32 |
|  |  |  | C-1-3 | 121,021,230 | 16.91 | 106,415,220 | 14.37 |
|  |  | Shoot | C-2-1 | 120,032,546 | 16.77 | 105,545,859 | 14.25 |
|  |  |  | C-2-2 | 103,491,027 | 14.46 | 910,00,730 | 12.29 |
|  |  |  | C-2-3 | 126,755,433 | 17.71 | 111,457,363 | 15.05 |
|  | Cd | Root | C-3-1 | 100,987,313 | 14.11 | 887,991,89 | 11.99 |
|  |  |  | C-3-2 | 82,257,514 | 11.49 | 75,265,625 | 10.51 |
|  |  |  | C-3-3 | 110,617,638 | 15.45 | 101,215,138 | 14.14 |
|  |  | Shoot | C-4-1 | 104,117,927 | 14.54 | 95,267,903 | 13.31 |
|  |  |  | C-4-2 | 81,413,128 | 11.37 | 74,493,012 | 10.40 |
|  |  |  | C-4-3 | 84,060,258 | 11.74 | 739,150,54 | 9.98 |

**Table S2** Summary of the BUSCO evaluation

| **BUSCO category** | ***V. baoshanensis*** | ***V. inconspicua*** |
| --- | --- | --- |
| Complete BUSCOs | 2,074 (97.7%) | 1,696 (80.0%) |
| Complete Single-Copy BUSCOs | 236 (11.1%) | 606 (28.6%) |
| Complete Duplicated BUSCOs | 1,838 (86.6%) | 1,090 (51.4%) |
| Fragmented BUSCOs | 31 ( 1.4%) | 217 (10.2%) |
| Missing BUSCOs | 16 ( 0.7%) | 208 ( 9.8%) |

**Table S3** Statistics of up-regulated DEGs in the UPS pathway

|  | **VB/VI(Cd)** | | **VB/VI(CK)** | | **Cd/CK(VB)** | | **Cd/CK(VI)** | |
| --- | --- | --- | --- | --- | --- | --- | --- | --- |
|  | **Root** | **Shoot** | **Root** | **Shoot** | **Root** | **Shoot** | **Root** | **Shoot** |
| E2 | 2 | 2 | 3 | 4 | 0 | 5 | 0 | 1 |
| HECT | 1 | 0 | 0 | 2 | 1 | 0 | 5 | 1 |
| Ubox | 5 | 1 | 1 | 7 | 48 | 2 | 26 | 5 |
| RING finger | 21 | 12 | 15 | 26 | 47 | 14 | 51 | 5 |
| Fbox | 12 | 15 | 15 | 18 | 28 | 15 | 22 | 2 |
| proteasome | 4 | 1 | 2 | 0 | 1 | 0 | 1 | 0 |

VB, *V. baoshanensis*. VI, *V. inconspicua*. CK, control.

**Table S4** Statistics of down-regulated DEGs in the UPS pathway

|  | **VB/VI(Cd)** | | **VB/VI(CK)** | | **Cd/CK(VB)** | | **Cd/CK(VI)** | |
| --- | --- | --- | --- | --- | --- | --- | --- | --- |
|  | **Root** | **Shoot** | **Root** | **Shoot** | **Root** | **Shoot** | **Root** | **Shoot** |
| E2 | 2 | 1 | 2 | 1 | 1 | 0 | 2 | 0 |
| HECT | 0 | 0 | 0 | 1 | 0 | 0 | 0 | 0 |
| Ubox | 1 | 0 | 2 | 2 | 0 | 3 | 2 | 0 |
| RING finger | 11 | 14 | 11 | 13 | 18 | 3 | 31 | 7 |
| Fbox | 7 | 12 | 14 | 13 | 15 | 8 | 0 | 7 |
| proteasome | 1 | 2 | 2 | 2 | 1 | 0 | 1 | 0 |

VB, *V. baoshanensis.* VI, *V. inconspicua*. CK, control.

**Table S5** Statistics of up-regulated DEGs in the sucrose metabolism pathway.

|  | **VB/VI(Cd)** | | **VB/VI(CK)** | | **Cd/CK(VB)** | | **Cd/CK(VI)** | |
| --- | --- | --- | --- | --- | --- | --- | --- | --- |
|  | **Root** | **Shoot** | **Root** | **Shoot** | **Root** | **Shoot** | **Root** | **Shoot** |
| AMY | 0 | 1 | 0 | 1 | 1 | 0 | 1 | 0 |
| bcsA | 1 | 0 | 1 | 0 | 2 | 1 | 4 | 2 |
| bglX | 2 | 1 | 1 | 1 | 2 | 0 | 1 | 1 |
| Callose synthase | 4 | 3 | 4 | 8 | 6 | 3 | 1 | 9 |
| endoglucanase | 1 | 1 | 3 | 2 | 1 | 5 | 0 | 2 |
| FRK | 0 | 0 | 0 | 0 | 1 | 0 | 3 | 0 |
| GYG1 | 2 | 0 | 0 | 0 | 7 | 1 | 1 | 0 |
| HXK | 1 | 0 | 1 | 1 | 0 | 0 | 1 | 0 |
| INV | 1 | 1 | 1 | 1 | 2 | 0 | 8 | 3 |
| malZ | 0 | 2 | 1 | 1 | 0 | 1 | 0 | 2 |
| TPP | 0 | 1 | 1 | 1 | 4 | 4 | 11 | 1 |
| SPP | 2 | 1 | 1 | 3 | 0 | 0 | 0 | 1 |
| SPS | 1 | 0 | 1 | 1 | 0 | 0 | 0 | 0 |
| TPS | 1 | 1 | 0 | 2 | 0 | 0 | 0 | 0 |
| TREH | 0 | 2 | 1 | 3 | 7 | 0 | 11 | 1 |

VB, *V. baoshanensis*. VI, *V. inconspicua*. CK, control.

**Table S6** Statistics of down-regulated DEGs in the sucrose metabolism pathway.

|  | **VB/VI(Cd)** | | **VB/VI(CK)** | | **Cd/CK(VB)** | | **Cd/CK(VI)** | |
| --- | --- | --- | --- | --- | --- | --- | --- | --- |
|  | **Root** | **Shoot** | **Root** | **Shoot** | **Root** | **Shoot** | **Root** | **Shoot** |
| AMY | 4 | 2 | 3 | 2 | 1 | 2 | 4 | 0 |
| bcsA | 0 | 1 | 1 | 0 | 0 | 1 | 0 | 0 |
| bglX | 0 | 0 | 0 | 0 | 3 | 0 | 0 | 0 |
| Callose synthase | 0 | 0 | 0 | 0 | 0 | 0 | 2 | 0 |
| endoglucanase | 1 | 0 | 0 | 0 | 8 | 0 | 5 | 0 |
| FRK | 2 | 2 | 2 | 1 | 0 | 0 | 0 | 0 |
| GYG1 | 0 | 1 | 1 | 4 | 0 | 1 | 1 | 2 |
| HXK | 0 | 0 | 0 | 0 | 1 | 0 | 1 | 0 |
| INV | 1 | 1 | 0 | 0 | 1 | 3 | 0 | 1 |
| malZ | 1 | 0 | 0 | 0 | 7 | 0 | 0 | 0 |
| TPP | 0 | 0 | 0 | 0 | 0 | 0 | 0 | 2 |
| SPP | 0 | 0 | 0 | 0 | 0 | 0 | 0 | 0 |
| SPS | 0 | 0 | 1 | 0 | 0 | 2 | 5 | 1 |
| TPS | 0 | 0 | 0 | 0 | 0 | 0 | 1 | 0 |
| TREH | 0 | 0 | 0 | 0 | 0 | 0 | 0 | 0 |

VB, *V. baoshanensis*. VI, *V. inconspicua*. CK, control.

**Table S7** Statistics of up-regulated DEGs encoding transporters

|  | **VB/VI(Cd)** | | **VB/VI(CK)** | | **Cd/CK(VB)** | | **Cd/CK(VI)** | |
| --- | --- | --- | --- | --- | --- | --- | --- | --- |
|  | **Root** | **Shoot** | **Root** | **Shoot** | **Root** | **Shoot** | **Root** | **Shoot** |
| ZIP | 4 | 2 | 5 | 1 | 1 | 14 | 3 | 2 |
| YSL | 0 | 0 | 0 | 0 | 0 | 1 | 7 | 3 |
| ABC | 11 | 12 | 8 | 15 | 28 | 9 | 21 | 11 |
| MTP | 0 | 1 | 2 | 0 | 2 | 2 | 3 | 0 |
| nramp | 1 | 1 | 1 | 1 | 2 | 2 | 2 | 1 |
| MATE | 0 | 1 | 0 | 1 | 8 | 0 | 5 | 4 |
| HIPP | 4 | 4 | 4 | 3 | 2 | 2 | 4 | 0 |
| HMA | 1 | 1 | 2 | 2 | 0 | 1 | 4 | 3 |
| CTR | 1 | 0 | 1 | 0 | 0 | 2 | 0 | 0 |
| CaCA | 0 | 0 | 0 | 0 | 0 | 0 | 2 | 0 |

VB, *V. baoshanensis*. VI, *V. inconspicua*. CK, control.

**Table S8** Statistics of down-regulated DEGs encoding transporters

|  | **VB/VI(Cd)** | | **VB/VI(CK)** | | **Cd/CK(VB)** | | **Cd/CK(VI)** | |
| --- | --- | --- | --- | --- | --- | --- | --- | --- |
|  | **Root** | **Shoot** | **Root** | **Shoot** | **Root** | **Shoot** | **Root** | **Shoot** |
| ZIP | 0 | 0 | 0 | 2 | 0 | 0 | 0 | 0 |
| YSL | 2 | 1 | 0 | 0 | 1 | 2 | 1 | 0 |
| ABC | 3 | 0 | 4 | 2 | 7 | 3 | 18 | 5 |
| MTP | 2 | 3 | 1 | 2 | 2 | 0 | 1 | 0 |
| nramp | 0 | 0 | 0 | 0 | 2 | 1 | 0 | 0 |
| MATE | 0 | 0 | 1 | 0 | 1 | 2 | 3 | 1 |
| HIPP | 0 | 3 | 0 | 5 | 4 | 3 | 5 | 4 |
| HMA | 0 | 0 | 0 | 0 | 0 | 0 | 0 | 0 |
| CTR | 1 | 1 | 1 | 1 | 0 | 0 | 0 | 0 |
| CaCA | 2 | 2 | 1 | 1 | 6 | 0 | 2 | 4 |

VB, *V. baoshanensis*. VI, *V. inconspicua*. CK, control.


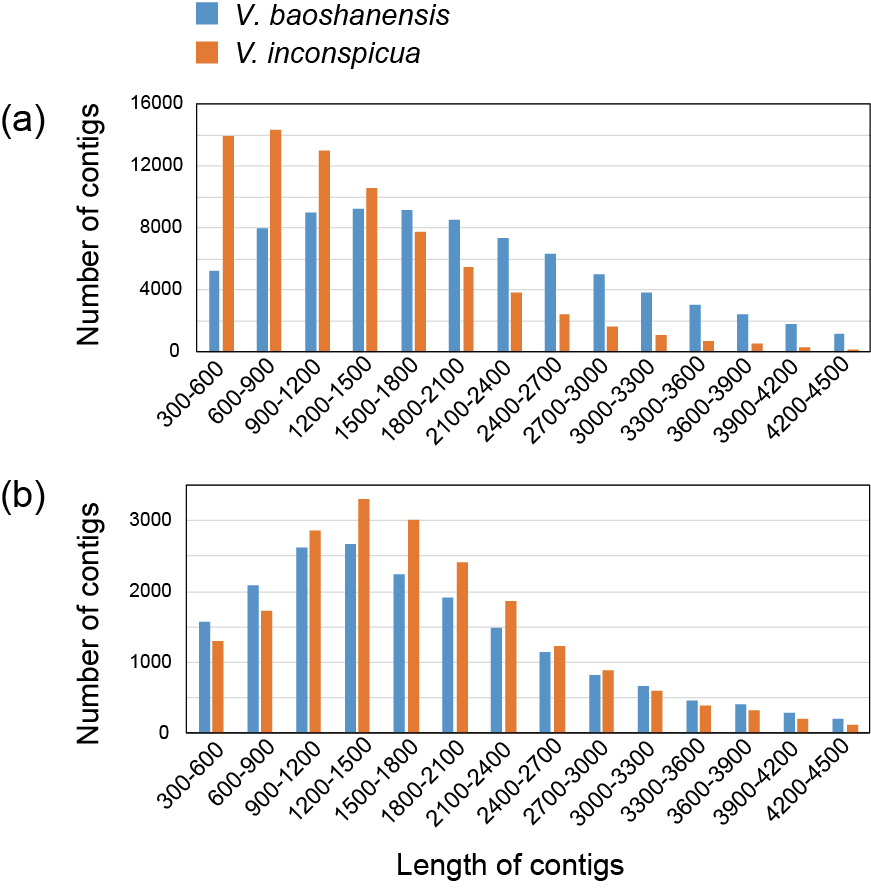


**Figure S1** Length distribution of entire transcripts (a) and orthologous transcripts (b) of *V. baoshanensis* and *V. inconspicua*.


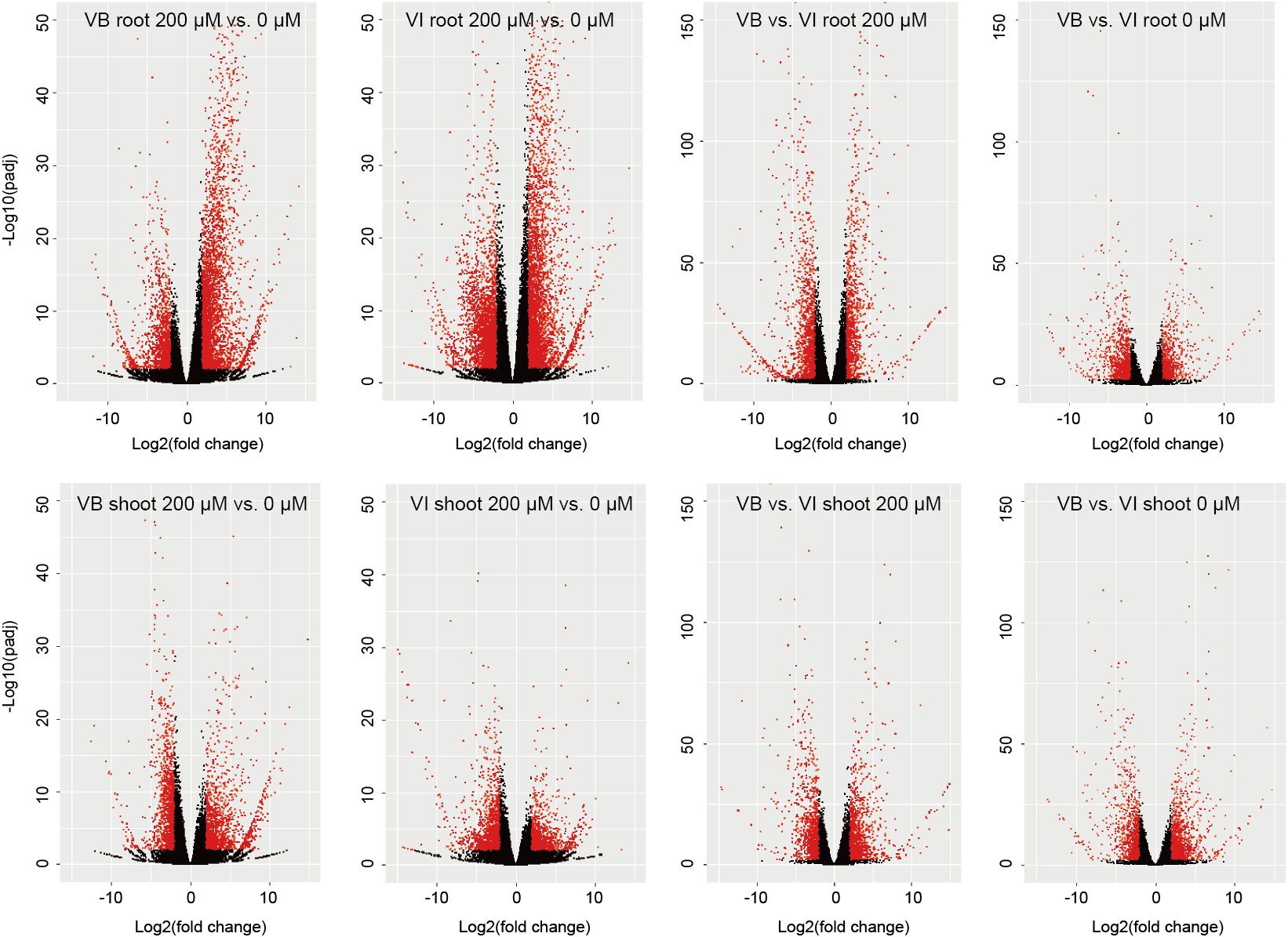


**Figure S2** Volcano plots of gene transcription-level changes in the two *Viola* species with different Cd treatments (from 0 µM to 100 µM). VB: *Viola baoshanensis,* VI: *V. inconspicua*. Red dots represent genes with significant differential transcription (padj < 0.01, |Log_2_(fold chang)| > 2). For the comparisons of interspecies, reads counting and differential transcription analysis were restricted to the orthologous gene sets annotated in both *Viola* species.


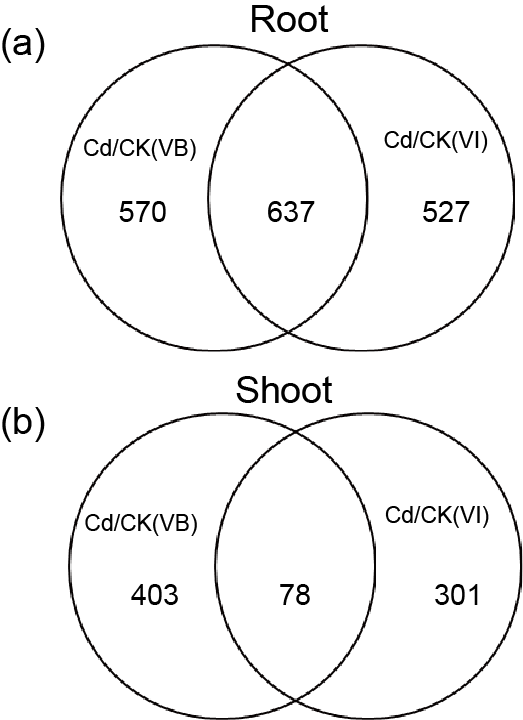


**Figure S3** Venn diagram of specific and commonly up-regulated transcripts of *V. baoshanensis* and *V. inconspicua* in response to Cd stress in roots (a) and shoots (b).


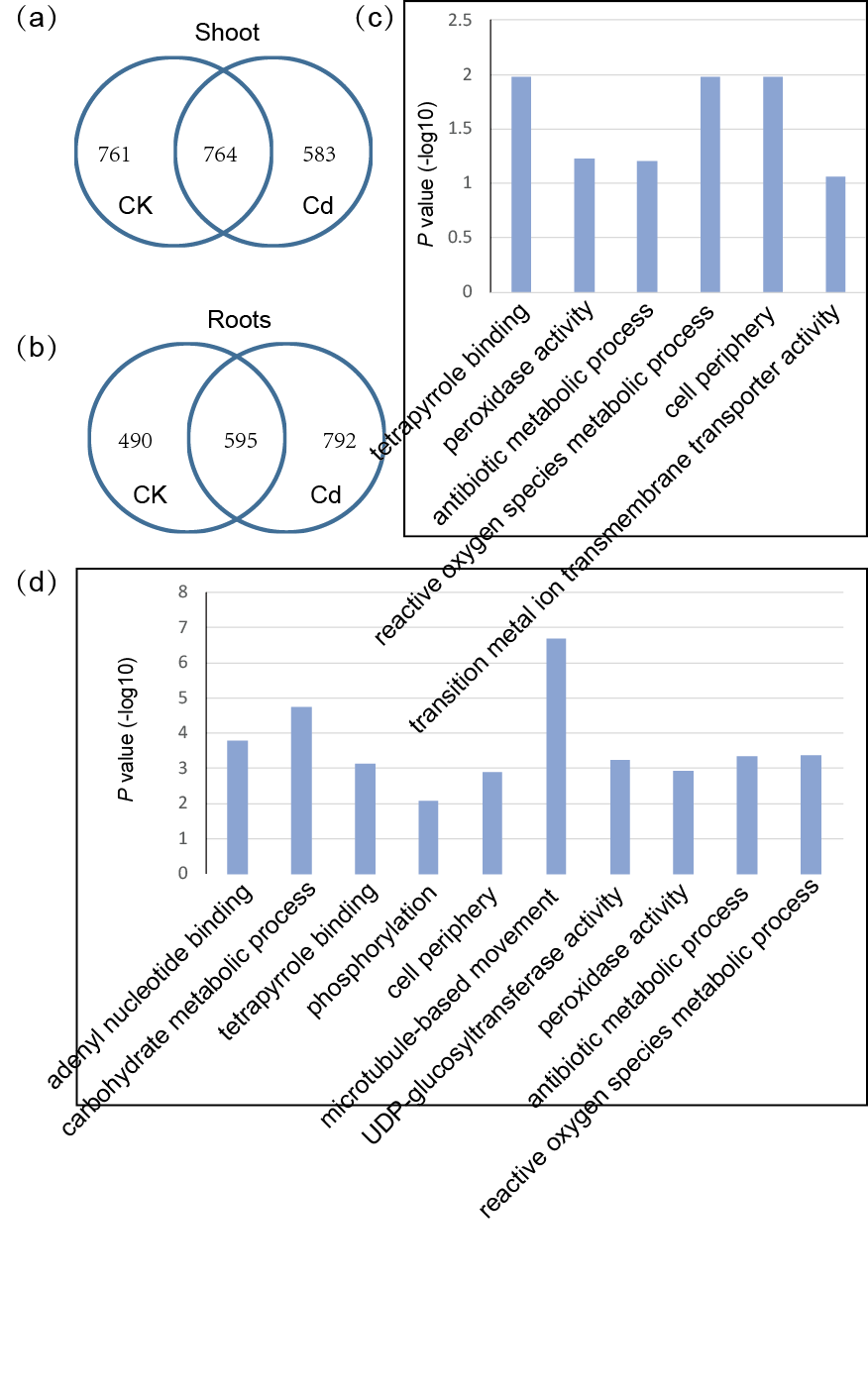


**Figure S4** Summary of significantly up-regulated genes in *V. baoshanensis* compared with *V. inconspicua*. Venn diagrams present the overlaps of up-regulated DEGs in roots (**a**) and shoots (**b**) between the Cd treatments (Cd) and controls (CK). GO enrichment analysis was performed to annotate the up-regulated DEGs in roots (**c**) and shoots (**d**).


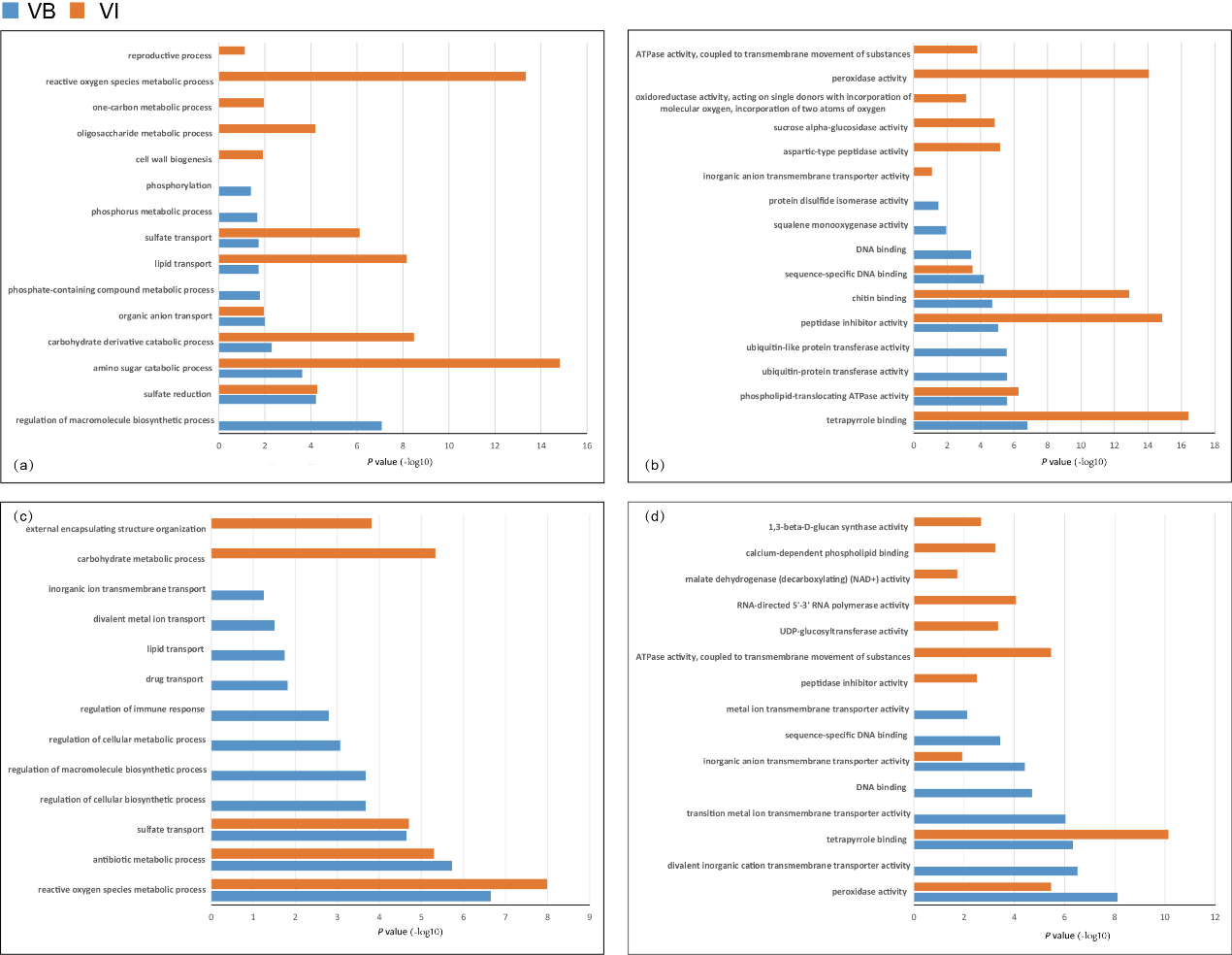


**Figure S5** GO enrichment of up-regulated DEGs in *V. baoshanensis* and *V. inconspicua* under Cd stress. Significance of enriched GO terms of the biological process (**a** and **c**) and the molecular function (**b** and **d**) were summarized for up-regulated DEGs between the Cd treatments (Cd) and controls (CK) in roots (**a** and **b**) and shoots (**c** and **d**). VB: *V. baoshanensis,* VI: *V. inconspicua.*


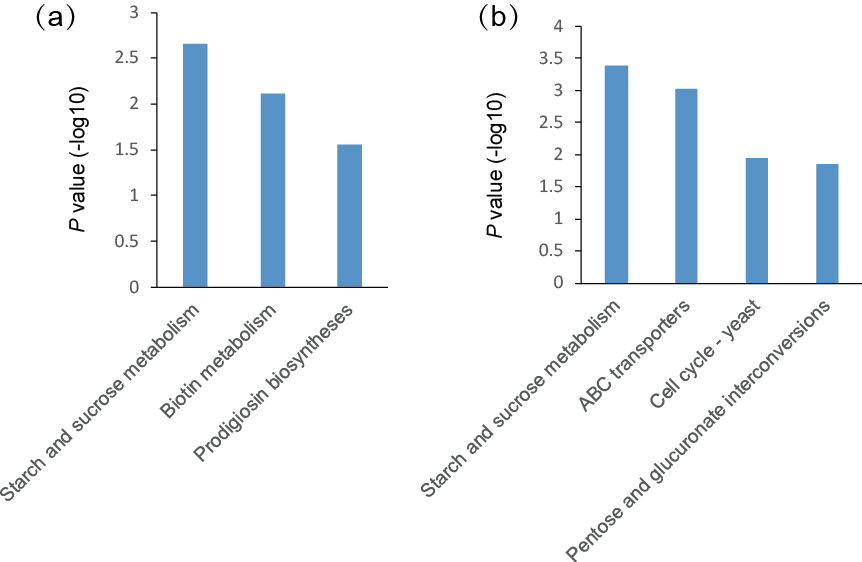


**Figure S6** KEGG enrichment of up-regulated DEGs in roots (**c**) and shoots (**d**) of *V. baoshanensis* compared with *V. inconspicua*.


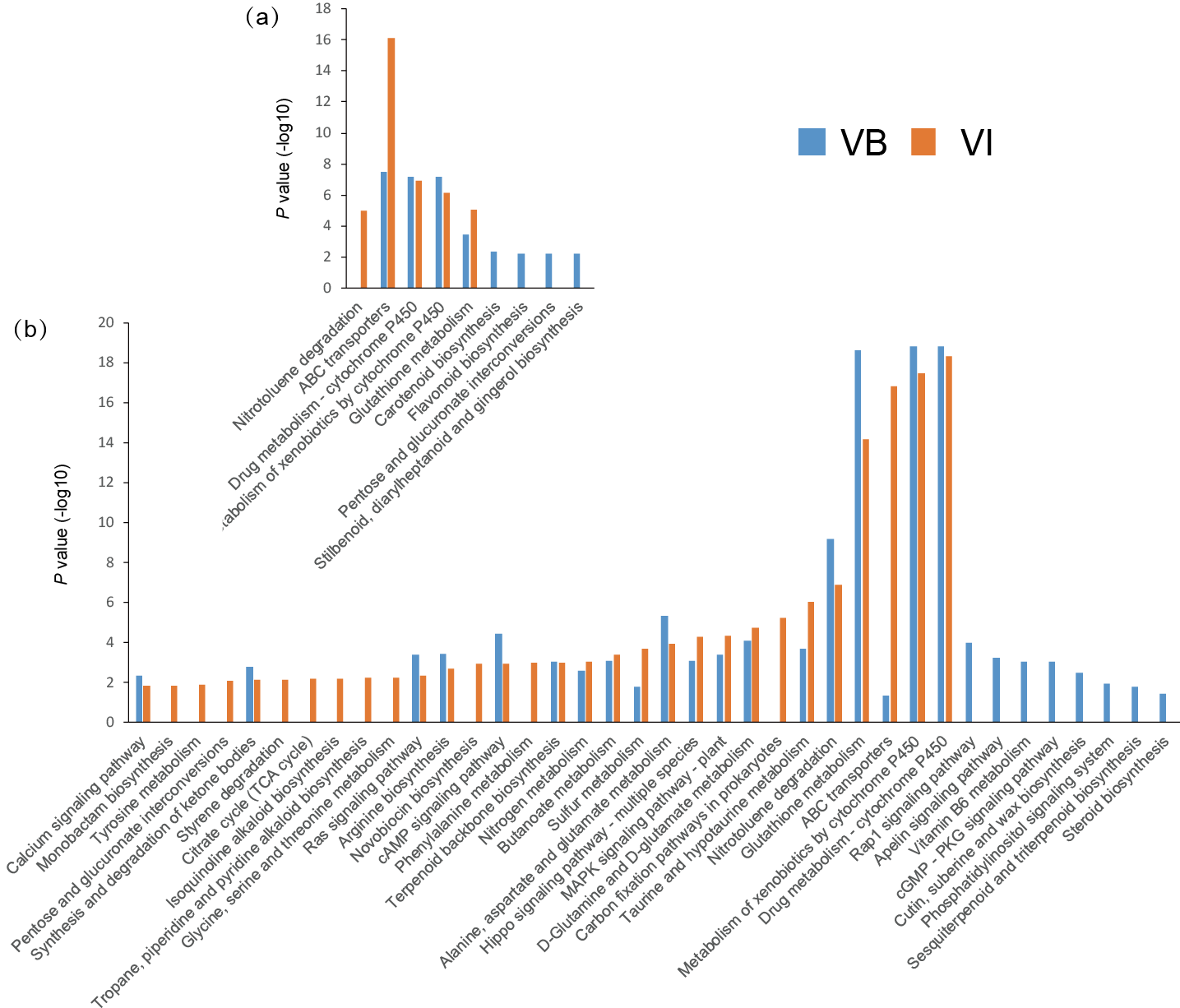


**Figure S7** KEGG enrichment of up-regulated DEGs in *V. baoshanensis* and *V. inconspicua*. The enriched KEGG terms of up-regulated DEGs were compared between the Cd treatments (Cd) and controls (CK) in roots (**a**) and shoots (**b**). VB: *V. baoshanensis,* VI: *V. inconspicua.*
